# A Functional Variant Alters the Binding of *Bone morphogenetic protein 2* to the Transcription Factor NF-κB to Regulate *Bone morphogenetic protein 2* Gene Expression and Chicken Abdominal Fat Deposition

**DOI:** 10.3390/ani13213401

**Published:** 2023-11-02

**Authors:** Meng Yuan, Xin Liu, Mengdie Wang, Ziwei Li, Hui Li, Li Leng, Shouzhi Wang

**Affiliations:** 1 Key Laboratory of Chicken Genetics and Breeding, Ministry of Agriculture and Rural Affairs, Harbin 150030, China; yuanmeng1501@163.com (M.Y.); 18845047068@163.com (X.L.); wangmengdie0424@163.com (M.W.); 18846813905@163.com (Z.L.); lihui@neau.edu.cn (H.L.); 2 Key Laboratory of Animal Genetics, Breeding and Reproduction, Education Department of Heilongjiang Province, Harbin 150030, China; 3 College of Animal Science and Technology, Northeast Agricultural University, Harbin 150030, China

**Keywords:** *Bone morphogenetic protein 2* (*BMP2*), chicken, functional variation, transcriptional regulation

## Abstract

**Simple Summary:**

*Bone morphogenetic protein 2* (*BMP2*) is crucial in numerous biological processes including osteogenesis, adipogenesis, and myogenesis. Our previous study has demonstrated that a 12-base pair (bp) insertion/deletion (InDel) variant (namely g.14798187_14798188insTCCCTGCCCCCT) within intron 2 of the chicken *BMP2* gene was significantly associated with chicken abdominal fat weight and abdominal fat percentage. However, the molecular mechanism underlying this association remains elusive. This study aimed to investigate whether the 12-bp InDel variant is a functional marker that affects the expression of the chicken *BMP2* gene and potential regulatory mechanism using both a dual-luciferase reporter assay and electrophoretic mobility shift assay in vitro. The results revealed that the 12-bp InDel chicken *BMP2* gene variant is a functional variant affecting fat deposition in chickens, which may also partially regulate *BMP2* gene expression by affecting the binding of transcription factor *NF-κB* to the *BMP2* gene. The findings will offer a potential functional molecular marker for improving the abdominal fat content of chickens in molecular breeding such as genome editing and genomic selection. Furthermore, the findings will also contribute to the understanding of the underlying roles of the *BMP2* gene in the growth and development of chicken adipose tissues.

**Abstract:**

In this study, we employed a dual-luciferase reporter assay and electrophoretic mobility shift analysis (EMSA) in vitro to explore whether a 12-base pair (bp) insertion/deletion (InDel) variant (namely g.14798187_14798188insTCCCTGCCCCCT) within intron 2 of the chicken *BMP2* gene, which was significantly associated with chicken abdominal fat weight and abdominal fat percentage, is a functional marker and its potential regulatory mechanism. The reporter analysis demonstrated that the luciferase activity of the deletion allele was extremely significantly higher than that of the insertion allele (*p* < 0.01). A bioinformatics analysis revealed that compared to the deletion allele, the insertion allele created a transcription factor binding site of *nuclear factor-kappa B* (*NF-κB*), which exhibited an inhibitory effect on fat deposition. A dual-luciferase reporter assay demonstrated that the inhibitory effect of *NF-κB* on the deletion allele was stronger than that on the insertion allele. EMSA indicated that the binding affinity of *NF-κB* for the insertion allele was stronger than that for the deletion allele. In conclusion, the 12-bp InDel chicken *BMP2* gene variant is a functional variant affecting fat deposition in chickens, which may partially regulate *BMP2* gene expression by affecting the binding of transcription factor *NF-κB* to the *BMP2* gene.

## 1. Introduction

In recent decades, continuous selective breeding has substantially increased the growth rate of broilers [1]. However, rapid growth in broilers causes excessive abdominal fat deposition [2], which not only reduces feed utilization, immunity, and reproductive performance, but also increases the fat content of chicken meat, which increases the risk of cardiovascular diseases among consumers [3,4,5,6]. In addition, chicken abdominal fat plays a decisive role in chicken carcass quality. Hence, preventing excessive fat deposition in broilers, and improving feed utilization and carcass quality of broilers are of utmost importance in the poultry industry [7,8,9,10]. The selection of low-fat and grain-saving broilers has become a pivotal goal of broiler breeding worldwide [11].

*Bone morphogenetic protein 2* (*BMP2*) is a member of bone morphogenetic proteins that belongs to the transforming growth factor β superfamily. *BMP2* is a secreted protein with a highly conserved sequence [12]. It was unexpectedly discovered in 1993 that the *BMP2* gene not only differentiates pluripotent stem cells into bone cells but also differentiates them into adipocytes [13]. Subsequently, Devaney et al. reported a single nucleotide protein in the 3′ untranslated region of the human *BMP2* gene and confirmed that the variant is significantly related to the formation of human subcutaneous fat [14]. Several studies have demonstrated that *BMP2* can promote adipogenesis by enhancing the transcriptional activity of PPARγ [15,16].

Numerous studies have exhibited that *BMP2* plays an important role in regulating lipogenesis and lipid metabolism in agricultural animals. In mammals, *BMP2* promotes the differentiation of porcine preadipocytes [17,18] and regulates fat deposition in sheep tails to alter tail types [19,20,21,22]. The expression of *BMP2* is high in bovine adipose tissues, and the exogenous addition of *BMP2* can facilitate the proliferation of bovine preadipocytes [23]. In poultry, *BMP2* affects duck abdominal fat deposition and is regulated by related long non-coding RNA [24]. In our previous study, *BMP2* was highly expressed in chicken abdominal fat tissues, and a 12-base pair (bp) insertion/deletion (InDel) variant (named g.14798187_14798188insTCCCTGCCCCCT) within intron 2 of the *BMP2* gene was indicated to be significantly associated with abdominal fat weight (AFW) and percentage of abdominal fat (AFP) in chickens [25,26].

This study aimed to investigate whether the 12-bp InDel is a functional variant affecting the expression of the chicken *BMP2* gene and its potential regulatory mechanism employing a dual-luciferase reporter assay and electrophoretic mobility shift assay (EMSA) in vitro.

## 2. Materials and Methods

### 2.1. Statement

All animal experiments were conducted per the guidelines for the care and use of experimental animals formulated by the Ministry of Science and Technology of the People’s Republic of China (Approval number: 2006-398), and the study was approved by the Experimental Animal Management Committee of Northeast Agricultural University. The construction and transfection of plasmids were conducted per the directions of the Regulations on Safety Management of Agricultural Genetically Modified Organisms formulated by China (revised version 2017).

### 2.2. Experimental Animals and Sample Collection

Two broiler divergent selective lines for abdominal fat content (NEAUHLF) have been constructed at Northeast Agricultural University since 1996, using the Arbor Acres broilers as the breeding material via plasma very-low-density lipoprotein level and AFP [AFP = AFW/body weight at 7 weeks of age (BW_7_)] [27,28]. From the fourth generation, the difference in AFP between the two lines was extremely significant [28]. In the 23rd generation, the AFP of the fat line (6.12%) is 9.87 times higher than that of the lean line (0.62%) [7].

In the RNA experiment, we collected abdominal fat tissues from 7-week-old male chickens (fat line, *n* = 5; lean line, *n* = 5) of the 23rd generation of NEAUHLF. The samples were stored at −80 °C until they were removed for RNA extraction.

### 2.3. Construction of Luciferase Reporter Vector

Based on the chicken *BMP2* gene (NC_052533.1) in GenBank, we synthesized DNA fragments with and without 12-bp sequence for the InDel variant by GENEWIZ (Suzhou, China) and separately cloned the DNA fragment into the upstream of SV40 promoter of pGL3-promoter vector. The pGL3-promoter vectors containing deletion and insertion alleles were named pGL3-*BMP2*-DD and pGL3-*BMP2*-II, respectively.

### 2.4. Bioinformatics Analysis

To investigate the potential molecular mechanism of the 12-bp InDel variant of the *BMP2* gene with abdominal fat deposition, we analyzed the transcription factor binding site within the 24-bp centered on the variant using Alibaba (http://gene-regulation.com accessed on 5 November 2022).

### 2.5. Designing of Primers

Primer Premier 5.0 software (Premier, Palo Alto, CA, USA) was used to design primers for *nuclear factor-kappa B* (*NF-κB*) and *β-actin*, based on their GenBank accession numbers (Table 1). The primer of *NF-κB* was used to construct a eukaryotic expression vector, and *β-actin* was used in Western blotting as a load control.

### 2.6. Cell Culture

Two cell lines were used in this experiment: one was the DF1 cell line, which is widely used in chicken cell and molecular research [29,30], and the other was immortalized chicken preadipocyte cell line (ICP-1) [31]. The DF1 cell line was used in a dual-luciferase reporter assay, Western blot, and EMSA. The ICP-1 cell line was used in a dual-luciferase reporter assay. Cells were cultured in DMEM/F12 medium (Gibco, New York, NY, USA) supplemented with 10% fetal bovine serum (Gibco), 100 U/mL penicillin, and 100 mg/mL streptomycin (Beyotime Institute of Biotechnology, Shanghai, China) and incubated in an incubator at 37 °C with 90% relative humidity and 5% CO_2_.

### 2.7. Construction and Validation of Eukaryotic Expression Vector

The total RNA was extracted from chicken abdominal fat tissues using the Trizol method (Invitrogen, Carlsbad, CA, USA) and reverse-transcribed into complementary DNA (cDNA) (Takara, Dalian, China). Using mixed cDNA as a template, the coding sequence (CDS) fragment of *NF-κB* was amplified by PCR with Phanta^®^ Max Super-Fidelity DNA Polymerase (Vazyme biotech Co., Ltd., Nanjing, China) and the primers, which are provided in Table 1. *NF-κB* expression plasmid was constructed by cloning the CDS fragment into pCMV-HA vector (Promega, Madison, WI, USA) using Cloning and Expression II one-step cloning kit (Vazyme Biotech Co, Ltd.). Following double digestion with *EcoR*I and *Xho*I (Takara), the recombinant plasmid was named pCMV-*NF-κB*.

To verify whether the *NF-κB* eukaryotic expression vector can be expressed normally, pCMV-*NF-κB* and pCMV-HA were separately transferred into chicken DF1 cells, and the total cell proteins was extracted 48 h later. The total cell proteins and 6× denaturing loading buffer were mixed and boiled for 5 min. Subsequently, the mixture was separated using 12% sodium dodecyl sulfate-polyacrylamide gel electrophoresis and transferred to a nitrocellulose filter membrane (Biosharp, Hefei, China). Western blotting was conducted by an antibody that recognizes HA-tag (1:1000; ZSGB-BIO, Beijing, China), and a secondary horseradish peroxide-conjugated antibody was added to enhance chemiluminescence (ZSGB-BIO).

### 2.8. Luciferase Reporter Assay

DF1 and ICP-1 cells were inoculated in 12-well plates, respectively, to transfect the luciferase report plasmids. After washing with phosphate-buffered saline at 70–80% confluence, different allele vectors (pGL3-*BMP2*-II and pGL3-*BMP2*-DD) or the pGL3-promoter vector, and the pRL-TK Renilla luciferase vector (Promega) were co-transfected into the cells using Lipofectamine 2000 (Invitrogen). DF1 and ICP-1 cells were inoculated in 24-well plates to detect the effect of *NF-κB* on the activity of pGL3-*BMP2*-II and pGL3-*BMP2*-DD reporter vectors, respectively. When the fusion rate reached 70–80%, pGL3-*BMP2*-II or pGL3-*BMP2*-DD, pCMV-*NF-κB* expression vector or pCMV-HA, and pRL-TK Renilla luciferase vector were co-transfected into cells using Lipofectamine 2000. The pRL-TK Renilla luciferase vector was used as an internal control. Following transfection, the cells were cultured in Opti-MEM^®^ for 6 h and further replaced with DMEM/F12 medium supplemented with 10% fetal bovine serum. Following 48 h of transfection, cells were collected, and the activities of Renilla luciferase (internal reference) and firefly luciferase were measured per the instructions of the dual-luciferase reporter assay system (Promega) [32]. Firefly luciferase activity was normalized to that of Renilla luciferase [33]. The reporter gene activity of different alleles was analyzed and compared.

### 2.9. Electrophoretic Mobility Shift Analysis

Biotin-labeled probes were prepared as indicated in Table 2. pCMV-*NF-κB* was transferred into DF1 cells to prepare the nuclear extracts interacting with the biotin probes. After 48 h, the nuclear extracts were collected using NE-PER nuclear and cytoplamic extraction reagents (Thermo, Waltham, MA, USA). The nuclear extracts and biotin-labeled DNA probes were incubated at room temperature for 20 min and then separated by electrophoresis on a 5% non-denaturing polyacrylamide gel with 0.5× Tris-borate ethylenediaminetetraacetic acid running buffer (Beyotime Institute of Biotechnology). DNA–protein complexes were transferred to the positively charged nylon film (Beyotime Institute of Biotechnology) and further cross-linked with a UV cross-linker agent for 1 min. The signal was detected following the manufacturer’s instructions provided with the LightShift chemiluminescence EMSA kit (Thermo). For the competition assay, the nuclear extracts and unlabeled probes were incubated at room temperature for 10 min prior to the addition of biotin-labeled oligonucleotides. The EMSA experiment was repeated twice.

### 2.10. Statistical Analysis

The experimental data were expressed as mean ± standard deviation (SD). JMP 11.0 (SAS Inst. Inc., Cary, NC, USA) was employed to compare the differences between the two groups using Student’s *t*-test. *p* < 0.05 or *p* < 0.01 was considered statistically significant or extremely significant, respectively.

## 3. Results

### 3.1. Luciferase Reporter Assay

**Figure 1:**
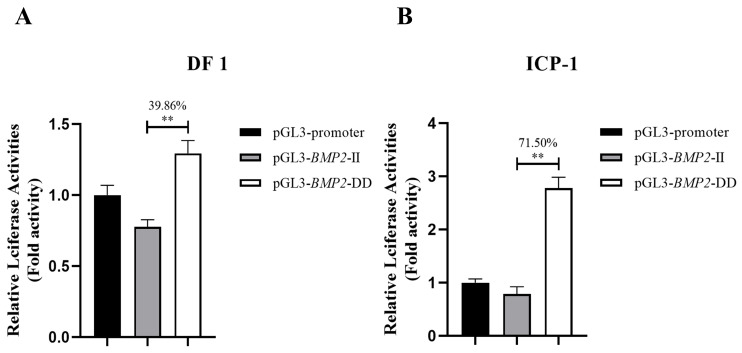
Luciferase activity of different alleles of *Bone morphogenetic protein 2* (*BMP2*) gene 12-base pair (bp) insertion/deletion (InDel) in DF1 and ICP-1 cells. (**A**) luciferase activity assay in DF1 cells. (**B**) luciferase activity assay in ICP-1 cells. Following 48 h of transfection, cells were collected, and the activities of Renilla luciferase (internal reference) and firefly luciferase were measured per the instructions of the dual-luciferase reporter assay system (Promega) [32]. The difference in values of relative luminous units (RLUs) was compared between different alleles of the 12-bp InDel and the pGL3-promoter vector. Results are demonstrated as fold change over pGL3-promoter activity. Values are indicated as the mean ± SD (*n* = 3). Note: ** indicates an extremely significant difference (*p* < 0.01).

Dual-luciferase reporter plasmids with different alleles (pGL3-*BMP2*-DD and pGL3-*BMP2*-II) were constructed to determine whether the 12-bp InDel variant of the chicken *BMP2* gene is functional. Further, pGL3-*BMP2*-DD and pGL3-*BMP2*-II were transfected into chicken DF1 and ICP-1 cells, respectively. The results demonstrated a significant difference in the dual-luciferase reporter activity of different alleles in two cell lines, and the activity of pGL3-*BMP2*-DD was extremely significantly higher than that of pGL3-*BMP2*-II (*p* < 0.01, Figure 1).

### 3.2. Bioinformatics Analysis

**Figure 2:**
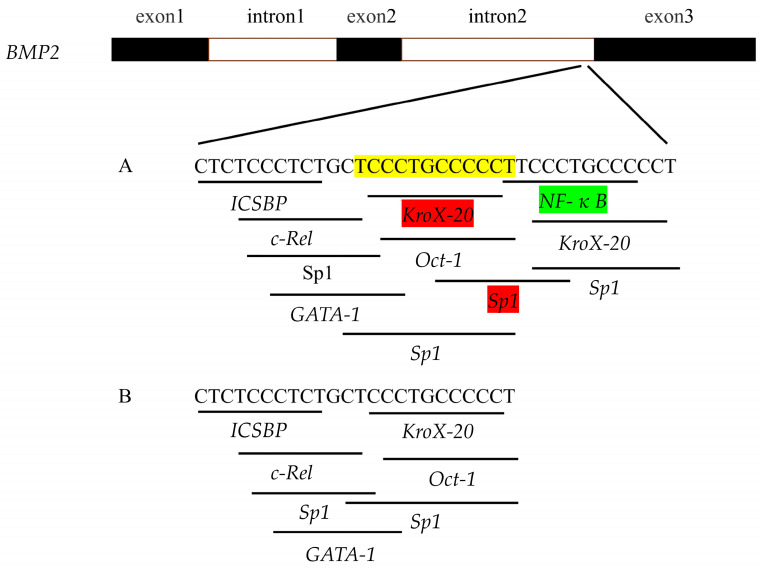
Changes in transcription factor binding sites at the 12-bp InDel variant of *BMP2* gene. (**A**) the insertion allele. (**B**) the deletion allele. Inserted sequence is indicated in yellow. Increased number of transcription factors are indicated in red. Created transcription factors are indicated in green.

We predicted the impact of the 12-bp InDel variant of the *BMP2* gene on transcription factor binding sites using Alibaba online software (http://gene-regulation.com accessed on 5 November 2022). The results demonstrated that there were many transcription factor binding sites for different alleles, such as *c-Rel*, *GATA binding protein-1*, *Octamer transcription factor 1* (*Oct-1*), *Specific protein 1* (*Sp1*), *Early growth response protein 2* (*Krox-20*), etc., (Figure 2). Compared with the deletion allele, the insertion allele not only created a transcription factor binding site of *NF-κB* but also increased the number of transcription factor binding sites such as *Krox-20* and *Sp1*. A great deal of evidence indicates that *NF-κB* plays an inhibitory role in fat deposition [33,34,35,36] and that *Krox-20* and *Sp1* promote adipogenesis [37,38,39,40,41]. Additionally, studies on the activity of different alleles implied that the insertion allele may bind to the inhibitory transcription factor to reduce activity (Figure 1). Consequently, we hypothesized that transcription factor *NF-κB* created by the 12-bp InDel variant may regulate *BMP2* gene expression. To confirm this speculation, we subsequently conducted a reporter assay and EMSA in vitro.

### 3.3. Construction of Transcription factor NF-κB Eukaryotic Expression Vector

**Figure 3:**
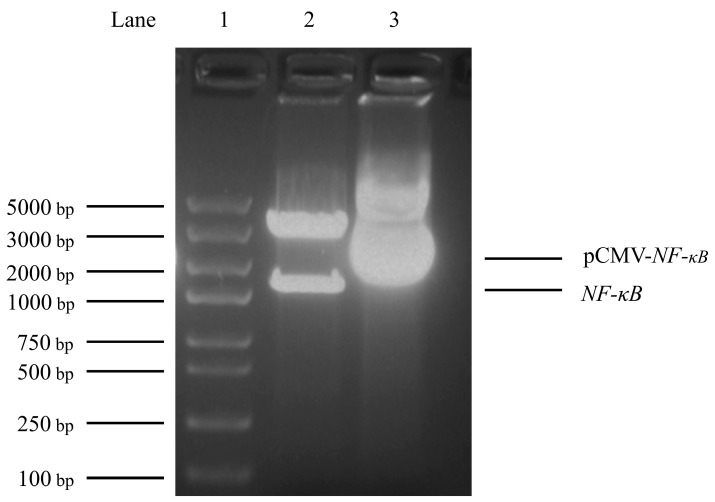
Identification of pCMV-*nuclear factor-kappa B* (*NF-κB*) plasmid by restriction enzyme digestion via *EcoR*I and *Xho*I double enzyme digestion. Lane 1: Marke. Lane 2: pCMV-*NF-κB* plasmid was digested with *EcoR*I and *Xho*I double enzymes. Lane 3: pCMV-*NF-κB* plasmid that was not digested with *EcoR*I and *Xho*I double enzymes.

**Figure 4:**
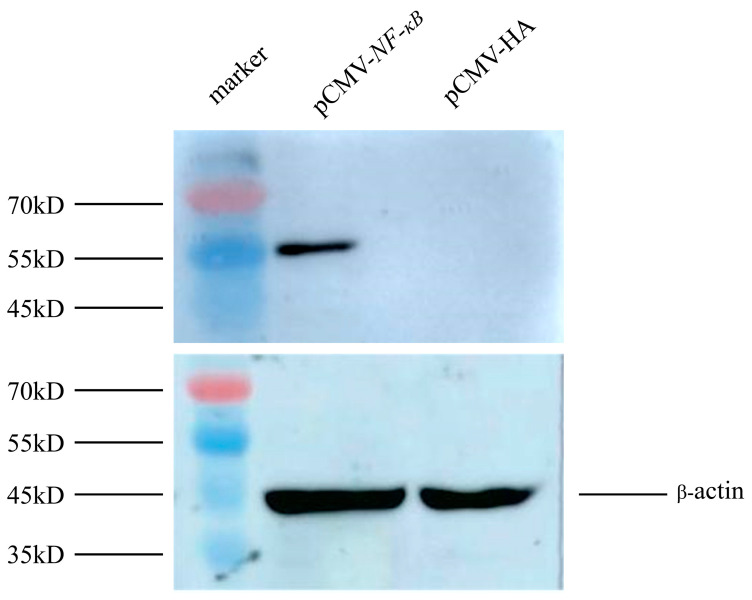
Western blot used to confirm the protein expression of pCMV-*NF-κB* eukaryotic expression vector in DF1 cells.

To detect the impact of *NF-κB* on the *BMP2* gene, the eukaryotic expression plasmid pCMV-*NF-κB* was first constructed and verified using double-restriction enzyme digestion (Figure 3). Further, pCMV-*NF-κB* and pCMV-HA vectors were transferred into DF1 cells, respectively, and the total protein of cells was collected after 48 h. The HA-labeled antibody was used as the main antibody, and *β-actin* was used as an internal reference. The expression effect of *NF-κB* in cells was analyzed using Western blot. The results revealed that DF1 cells transfected with pCMV-*NF-κB* expressed a specific protein with a size similar to that of the expected target protein, whereas DF1 cells transfected with pCMV-HA did not express any proteins, suggesting that *NF-κB* can be overexpressed in DF1 cells (Figure 4).

### 3.4. Transcription Factor NF-κB Regulates the Expression of BMP2 Gene via the 12-bp InDel Variation

**Figure 5:**
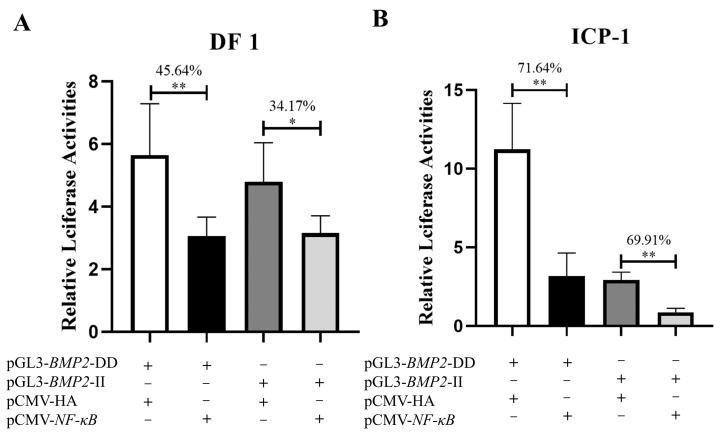
Regulation of *NF-κB* on *BMP2* gene 12-bp InDel variant in DF1 cells and ICP-1 cells. (**A**) DF1 cells were co-transfected with pGL3-*BMP2*-II or pGL3-*BMP2*-DD luciferase reporter vector and *NF-κB* eukaryotic expression vector. The transfection of pGL3-*BMP2*-II or pGL3-*BMP2*-DD luciferase reporter vector and pCMV-HA empty vector were used as control. (**B**) co-transfection of ICP-1 cells. Values are indicated as the mean ± SD (*n* = 6). Note: * indicates a significant difference (*p* < 0.05); ** indicates an extremely significant difference (*p* < 0.01).

We co-transfected pCMV-*NF-κB* or pCMV-HA, pGL3-*BMP2*-II or pGL3-*BMP2*-DD, and the pRL-TK Renilla luciferase vector into DF1 and ICP-1 cells to further examine the effect of *NF-κB* on the transcriptional activity of different alleles of the *BMP2* gene. The results demonstrated that the transcription factor *NF-κB* significantly inhibited the activity of pGL3-*BMP2*-II and pGL3-*BMP2*-DD luciferase reporter vectors in DF1 and ICP-1 cells (*p* < 0.05), and the inhibitory effect on pGL3-*BMP2*-DD was stronger than that on pGL3-*BMP2*-II (Figure 5).

### 3.5. Electrophoretic Mobility Shift Analysis

**Figure 6:**
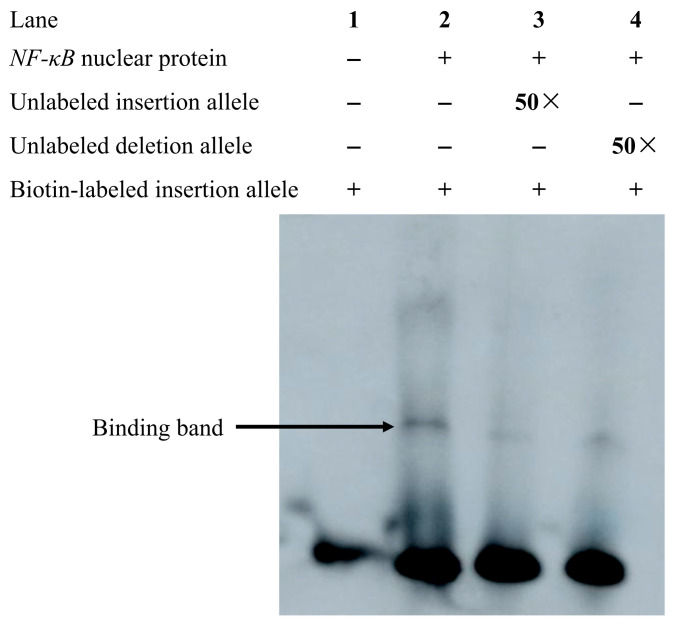
Analysis of binding of different alleles of *BMP2* gene with *NF-κB* using EMSA. Only biotin-labeled insertion allele probes were available (lane 1); binding of the biotin-labeled insertion allele probes to *NF-κB* nuclear protein (lane 2); 50-fold unlabeled insertion allele probes competed with biotin-labeled insertion allele probes (lane 3); 50-fold unlabeled deletion allele probes competed with biotin-labeled insertion allele probes (lane 4).

To ascertain whether the transcription factor *NF-κB* specifically binds to the transcription factor binding site on the chicken *BMP2* gene, biotin 5′ labeled single-stranded oligonucleotide probes at the *NF-κB* binding site were synthesized (Table 2). The single-stranded oligonucleotide probe was annealed to generate a double-stranded probe. The eukaryotic expression vector pCMV-*NF-κB* was transfected into DF1 cells, and the nuclear protein was extracted following 48 h for EMSA. The results demonstrated that biotin-labeled probes with the insertion allele and the DF1 nuclear protein overexpressing *NF-κB* produced a DNA-protein complex band (Figure 6; Lane 2). DNA-protein complex bands were weakened when 50-fold unlabeled insertion allele and deletion allele probes were added, respectively. However, the DNA-protein complex band with the 50-fold unlabeled insertion allele probes was more attenuated than the DNA-protein complex band with the 50-fold unlabeled deletion allele probes (Figure 6; Lanes 3, 4). Therefore, compared with the deletion allele, the insertion allele demonstrated stronger binding affinity with nuclear extracts rich in *NF-κB*. EMSA indicated that *NF-κB* can specifically bind to the variant.

## 4. Discussion

*BMP2* is widely involved in biological processes such as osteogenesis [42,43,44], adipogenesis [45,46], and myogenesis [47,48,49]. Our previous studies have demonstrated that the chicken *BMP2* gene is highly expressed in abdominal fat tissues, and a 12-bp InDel within intron 2 of the chicken *BMP2* gene was significantly associated with AFW and AFP, indicating that the *BMP2* gene may impact chicken abdominal fat deposition through the 12-bp InDel variant [25,26]. To investigate whether the variant is functional, we performed a luciferase reporter assay in vitro. Our study showed that in both DF1 and ICP-1 cells, the luciferase activity of the deletion allele was significantly higher than that of the insertion allele (*p* < 0.01, Figure 1), indicating that the variant may be functional and can regulate the *BMP2* gene expression in vitro. Notably, the luciferase reporter assay was performed in two different cell types (DF1 and ICP-1), which validated the reliability of the results.

A non-coding sequence, called an intron, can be transcribed into heterogeneous nuclear RNA (hnRNA) in eukaryotic DNA, albeit it is removed by splicing during the production of mRNA. Intronic variants of genes have been reported to regulate gene transcription by modifying transcription factor binding sites [33,50,51,52]. For instance, Meyer et al. discovered that two SNPs within intron 2 of the *fibroblast growth factor receptor 2* (*FGFR2*) gene regulate *FGFR2* gene expression by impacting the binding affinity of transcription factors *Oct-1*/*Runt-related transcription factor 2* and *CCAAT enhancer binding proteins*, and thereby influence the risk of human breast cancer [53]. In addition, it has been reported that a G-to-A variant within intron 3 of the *insulin-like growth factor II* (*IGF2*) gene elevates *IGF2* expression in skeletal muscle and lean meat yield by affecting the transcription factor binding site of *Zinc finger, BED-type containing 6* [54,55,56]. Transcription factors, which operate as transacting factors, are the primary regulators of gene expression. They regulate the transcription of target genes by binding with DNA [57,58]. We used bioinformatics analysis to explore the molecular mechanism of the 12-bp InDel variant, which demonstrated that the insertion allele in intron 2 of chicken *BMP2* can generate transcription factor binding sites of *NF-κB*, *Krox-20*, and *Sp1* (Figure 2). *NF-κB* is a transcription factor that regulates the expression of various genes [33,59]. It is retained in the cytoplasm by interacting with inhibitory proteins of the inhibitory factor kappa (I*κ*B) family and is present in many different types of cells [33,60,61]. Some studies have indicated that *NF-κB* exhibits an inhibitory effect on fat deposition in mouse [34], humans [35,36], and chickens [33]. This implies that *NF-κB* may inhibit *BMP2* transcriptional activity by binding to the insertion allele, which is consistent with the fact that the insertion allele’s activity was lower than that of the deletion allele (Figure 1). Hence, we hypothesized that the variant affects *BMP2* gene transcription by altering the binding ability of the *BMP2* gene to the transcription factor *NF-κB*, thus regulating abdominal fat deposition in chicken.

In this study, we confirmed that *NF-κB* significantly inhibited the transcriptional efficiency of different alleles in vitro, and the inhibitory effect for the deletion allele was stronger than that for the insertion allele (Figure 5). EMSA revealed that *NF-κB* specifically bound to the *BMP2* gene and that the binding affinity of *NF-κB* for the insertion allele was stronger than that for the deletion allele (Figure 6), which indicated that the 12-bp InDel variant may regulate *BMP2* gene expression by altering the binding ability of *BMP2* to the transcription factor *NF-κB*, thereby regulating abdominal fat deposition in chickens. However, it should be noted that the EMSA results suggested that the inhibitory effect of *NF-κB* on the insertion allele is stronger than that on the deletion allele, which is inconsistent with the findings demonstrating the impact of *NF-κB* on the activity of different alleles (Figure 5). The discrepancy may be attributed to the following three reasons: First, transcription factors can regulate gene expression not only directly by interacting with regulatory elements but also indirectly by interacting with other regulators [62,63]. Thus, we hypothesized that even though the deletion allele does not have a binding site for *NF-κB*, it plays an indirect role in the regulation of different allele activities via its interaction with other unknown factors, thus inhibiting the activity of the deletion allele. Second, a bioinformatics analysis indicated that the insertion allele creates transcription factor *NF-κB* binding sites while increasing the number of transcription factor binding sites such as *Krox-20* and *Sp1* (Figure 2). *NF-κB* is involved in the negative regulation of fat deposition [33,34,35,36], whereas *Krox-20* and *Sp1* have a positive impact on adipogenesis [37,38,39,40]. Hence, the possibility that *Krox-20* and/or *Sp1* may also be involved in the regulation of the transcriptional activity of the insertion allele is not ruled out, causing less reduction in the activity of the insertion allele compared to the deletion allele (Figure 5). Third, the 12-bp InDel variant is located in intron 2, close to the exon, and therefore, it may affect the expression of the *BMP2* gene by altering the splicing site of the mRNA [64,65,66,67,68]. Altogether, further investigation is needed to completely unveil the regulatory mechanism of the 12-bp InDel variant of *BMP2* from two facets such as determining more transcription factors and alternative splicing.

## 5. Conclusions

The g.14798187_14798188insTCCCTGCCCCCT within intron 2 of the chicken *BMP2* gene is a functional variant which may at least partially regulate the expression of the *BMP2* gene by affecting the binding of transcription factor *NF-κB* to the *BMP2* gene. Our findings will provide a potential functional molecular marker for improving the abdominal fat content of chickens in molecular breeding such as genome editing and genomic selection. At the same time, our findings maybe contribute to the understanding of the underlying roles of the *BMP2* gene in the growth and development of chicken adipose tissues.

## Figures and Tables

**Table 1:** Primers used in this study.

GenBank Accession No.	Primer Name	Primer Sequence (5′-3′)	Purpose
NM_001396395.1	*NF-κB*-F	5′-CCGTATCTTCAAATCATTGAACAGCC-3′	Construction of eukaryotic expression vector
	*NF-κB*-R	5′-ATAGCCTTCTCCAGGAACAGACCATC-3′	
NM_205518.1	*β-actin*-F	5′-TGGCCATGGAGGCCCGAATTCCCAAAATGCCAACCCT-3′	Loading control
	*β-actin*-R	5′-CCGCGGCCGCGGTACCTCGAGACTGCCCAGAAAGTTGTG-3′	

**Table 2:** Probes used in this study.

Transcription Factor	Probes	Probe Sequence (5′-3′)
*NF-κB*	*BMP2*-DD-F	CTCCCTCTGCTCCCTGCCCCCT
	*BMP2*-DD-R	AGGGGGCAGGGAGCAGAGGGAG
	*BMP2*-II-F	CTCCCTCTGCTCCCTGCCCCCTTCCCTGCCCCCT
	*BMP2*-II-R	AGGGGGCAGGGAAGGGGGCAGGGAGCAGAGGGAG
	*BMP2*-B-II-F	CTCCCTCTGCTCCCTGCCCCCTTCCCTGCCCCCT
	*BMP2*-B-II-R	AGGGGGCAGGGAAGGGGGCAGGGAGCAGAGGGAG

Note: *Bone morphogenetic protein 2 (BMP2)*-DD: unlabeled deletion allele probes for the *BMP2* gene; *BMP2*-II: unlabeled insertion allele probes for the *BMP2* gene; *BMP2*-B-II: biotin-labeled insertion allele probes for the *BMP2* gene.

## Data Availability

Not applicable.
